# Efficacy evaluation of neoadjuvant chemotherapy in patients with HER2-low expression breast cancer: A real-world retrospective study

**DOI:** 10.3389/fonc.2022.999716

**Published:** 2022-12-19

**Authors:** Lingfeng Tang, Zhenghang Li, Linshan Jiang, Xiujie Shu, Yingkun Xu, Shengchun Liu

**Affiliations:** Department of Breast and Thyroid Surgery, the First Affiliated Hospital of Chongqing Medical University, Chongqing, China

**Keywords:** HER2-low, neoadjuvant chemotherapy, pathologic complete response, nomogram, targeted therapy

## Abstract

**Background:**

To characterize the clinicopathological features and evaluate the neoadjuvant chemotherapy (NACT) efficacy of patients with human epidermal growth factor receptor 2 (HER2)-low breast cancer.

**Methods:**

A total of 905 breast cancer patients who received 4 cycles of thrice-weekly standard NACT in the First Affiliated Hospital of Chongqing Medical University were retrospectively enrolled, including 685 cases with HER2-low expression and 220 cases with HER2-negative expression. Clinicopathological features were compared between patients with HER2-negative and HER2-low expression. Univariate and multivariate logistic regression analyses were used to find the independent factors of achieving a pathological complete response (pCR) after NACT.

**Results:**

There were significant differences in stage_N (*P* = 0.014), histological grade (*P* = 0.001), estrogen receptor (ER) status (*P* < 0.001), progesterone receptor (PgR) status (*P* < 0.001), NACT regimens (*P* = 0.032) and NACT efficacy (*P* = 0.037) between patients with HER2-negative and HER2-low expression breast cancer. In subgroup analysis, histological grade (*P* = 0.032), ER (*P* = 0.002), Ki-67 (*P* < 0.001) and HER2 status (*P* = 0.025) were independent predictors of achieving a pCR in ER-positive breast cancer. And the nomogram for pCR in ER-positive breast cancer showed great discriminatory ability with an AUC of 0.795. The calibration curve also showed that the predictive ability of the nomogram was a good fit to actual observations. Then, in the analysis of ER-negative breast cancer, only stage_N (*P* = 0.001) and Ki-67 (*P* = 0.018) were independent influencing factors of achieving a pCR in ER-negative breast cancer.

**Conclusion:**

HER2-low breast cancer was a different disease from HER2-negative breast cancer in clinicopathological features. Moreover, the NACT efficacy of HER2-low breast cancer patients was poorer.

## Introduction

Breast cancer is recognized as a highly heterogeneous disease, which was distinguished distinct pathological subtypes through the expression of hormone receptors (HR) and human epidermal growth factor receptor 2 (HER2) (1). HER2-enriched breast cancer has been reported to be associated with aggressive clinical features and a poor prognosis, nevertheless, due to the development of anti-HER2 agents the outcomes of HER2-enriched breast cancer patients were significantly improved (2–5). But the remaining 85% of breast cancers patients with HER2-low expression (immunohistochemistry (IHC) 1+ or IHC 2+, fluorescence *in situ* hybridization (FISH) non-amplified) or HER2-negative expression (IHC 0) failed to derive no benefit from the currently available anti-HER2 treatments (6, 7).

Neoadjuvant chemotherapy (NACT), which is utilized before surgery, is mainly used for the management of patients with locally advanced breast cancer. By killing active cancer cells, NACT can effectively reduce the clinical stage of breast cancer, making inoperable breast cancer operable breast cancer or increasing the chances of breast conservation (8). Meanwhile, many studies have demonstrated that patients who achieve a pathological complete response (pCR) after NACT seem to have improved long-term outcomes (9, 10). However, chemoresistance has always been a clinical problem in the treatment of breast cancer. Some studies have shown that high HER2 expression indicated high viability, proliferation and invasive ability in tumor cells, in addition, increased drug resistance mediated by HER2 expression was an important factor for the tumor malignancy and poor patient prognosis (11). In HER2-positive breast cancer, HER2/HER3 can up-regulate survivin *via* the PI3K/Akt pathway and confer paclitaxel resistance to tumor cells (12–14). Moreover, it has been reported that HER2 can activate calmodulin dependent protein kinases and Raf/MEK/ERK signaling pathway in gastric cancer cells and induce drug resistance (15). Recently, a phase II study about a novel antibody-drug-conjugate (ADC) in HR-positive, HER2-low expression advanced breast cancer patients reported promising preliminary results in terms of clinical activity and safety (16). Besides, trastuzumab deruxtecan also showed the therapeutic potential for HER2-low expression breast cancer patients (17).

With the development of this novel therapeutic strategy, HER2-low expression breast cancer may be recognized as a distinct clinical entity. This study compared the clinicopathological characteristics of patients with HER2-low or HER2-negative expression and established a nomogram based on the influential factors of NACT for predicting the probability of achieving pCR. Such a model would be useful in evaluating sensitivity to chemotherapy, which can provide a reference for the use of novel anti-HER2 agents in neoadjuvant therapy.

## Methods

### Population

**Figure 1:**
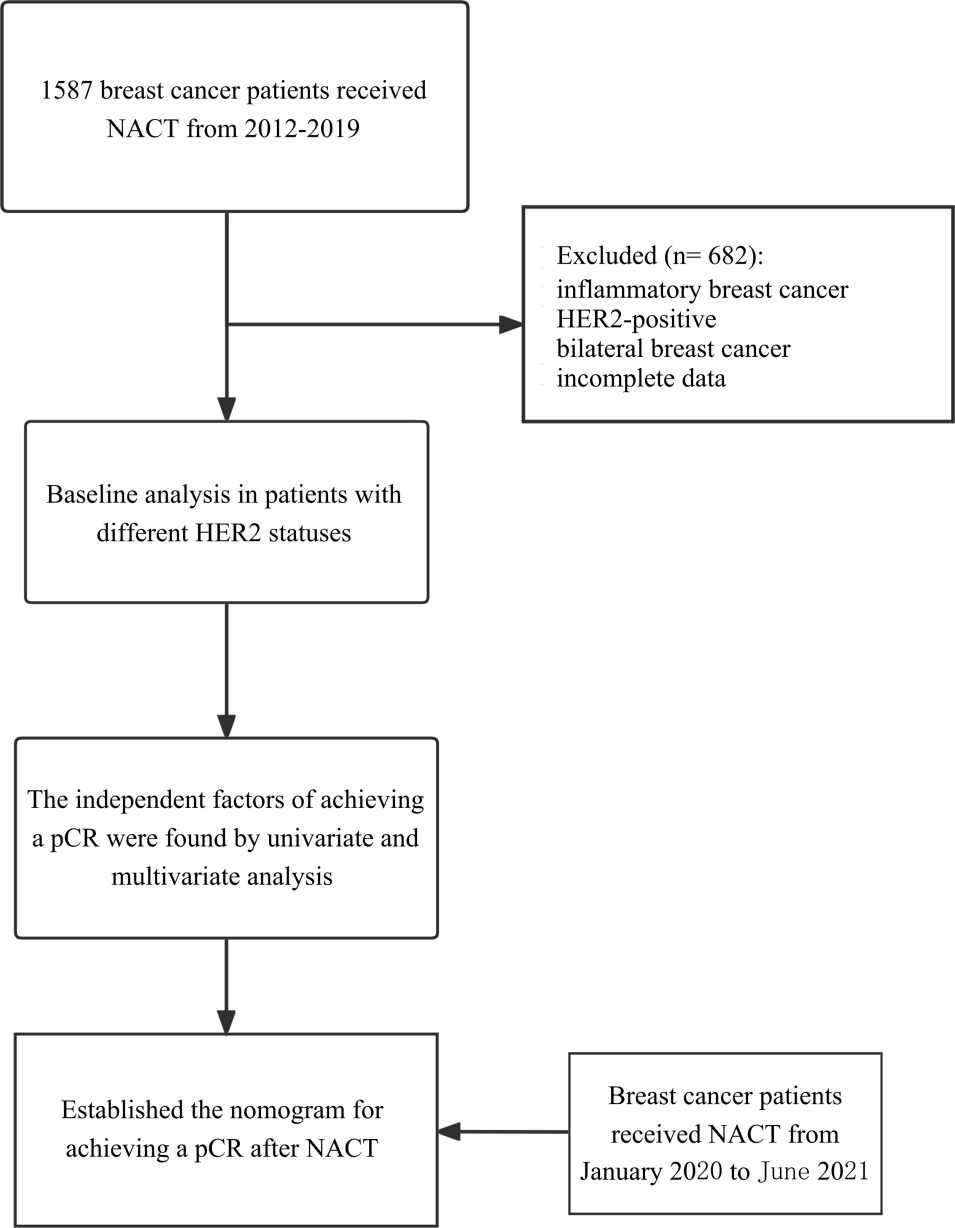
Study flowchart. Flow-chart shows the process of including patients in the study. NACT, neoadjuvant chemotherapy; pCR, pathologic complete response.

The database was reviewed to identify all patients diagnosed from the First Affiliated Hospital of Chongqing Medical University between 1 January 2012 and 31 December 2019. We used the following inclusion criteria: (I) female; (II) performed neoadjuvant chemotherapy; (III) invasive ductal breast cancer; and (IV) no anti-tumor treatment before NACT. The exclusion criteria were as follows: (I) inflammatory breast cancer; (II) HER2-enrich breast cancer (IHC 3+ or IHC 2+ with FISH amplified); (III) other primary tumors; (IV) bilateral breast cancer; and (V) incomplete data. A total of 905 eligible patients were ultimately included in this study. The database of patients diagnosed between 1 January 2020 and 31 June 2021 was collected according to the same standard, which would be used as the validation group of the nomogram (**Figure 1**).

All histological specimens were paraffin-embedded and evaluated by two skilled pathologists. This study was approved by the Ethics Committee of the First Affiliated Hospital of Chongqing Medical University (No. 2020-202). This article does not refer to the privacy of patients, so informed consent was exempted. All data were fully anonymized before we accessed them. The authors were not provided with information that could identify individual participants during or after data collection.

### Clinicopathologic analysis

Data on the medical history, concurrent diseases, age, menopausal status, histological grade, tumor size, lymph node (LN) status, HR status, Ki-67 index, and NACT regimens were estimated before NACT. Clinical assessments of the breast, including preoperative LN status, tumor size depended on MRI or breast ultrasonography. RECIST criteria were used for the clinical response evaluation (18). The estrogen receptor (ER), progesterone receptor (PgR) and Ki-67 status were evaluated by IHC of the pretreatment core biopsy specimens. The HER2-negative group consisted of the breast cancer patients with a completely negative HER2 staining (IHC score of 0) and the HER2-low group consisted of the breast cancer patients with low level of HER2 expression (IHC scores of 1+ and 2+ with FISH non-amplified). Cancers with 1–100% of cells positive for ER/PgR expression were considered ER-positive/PgR-positive. The Ki-67 index was defined as the percentage of the total number of tumor cells (at least 1000) with nuclear staining over 10 high powered fields (× 40).

### Treatment

The criteria for receiving neoadjuvant chemotherapy were as follows: patients the local stage of the disease was relatively late, such as patients with axillary lymph node metastasis or large mass or invasion of skin and chest wall, as well as patients who had a strong desire to do breast conserving surgery but did not meet the indication of breast conserving surgery when diagnosed.

NACT was given according to the local protocol and national guidelines. The treatments were predominantly anthracycline and taxane. The TEC (docetaxel 75 mg/m^2^, epirubicin 75 mg/m^2^, and cyclophosphamide 500 mg/m^2^) or EC (epirubicin 75 mg/m^2^, and cyclophosphamide 500 mg/m^2^) NACT regimens were administered every 3 weeks. After diagnosis, all patients started the first cycle of NACT in a week and received four cycles of NACT regimens we evaluated the clinical response.

### Objective

For all patients enrolled, mastectomy or breast conserving surgery (NACT for breast conservation) plus axillary lymphadenectomy was the basic surgical treatment after 4-cycle NACT. Two pathologists blindly and independently diagnosed all resected breast and lymph node specimens. Then, pCR was defined as no residual invasive cancer in the breast or evidence of disease in the axillary lymph nodes (ypT0ypN0) after NACT. In this study, we took pCR as our observation objective.

### Statistical methods

Statistical analysis was performed by R software (Version 4.2.0) and SPSS (Version 25.0). Categorical variables were compared using the chi-squared test or Fisher’s exact test. Then, univariate and multivariate logistic regression analyses were used to screen out the independent predictors. To quantify the discrimination performance of the nomogram, Harrell’s C-index was measured. The intolerant abilities of the model were assessed by measuring the area under the receiver operating characteristic (ROC) curve. Calibration curves were plotted to assess the calibration of the nomogram (19). In this case, the calibration is the agreement between the frequencies of the observed outcomes and the probabilities predicted by the model. *P* < 0.05 was defined as statistically significance.

## Results

### Baseline patient characteristics based on HER2 status

A total of 905 patients with HER2-low expression or HER2-negative breast cancer who received NACT were identified (mean age 49.2 ± 9.5 years (range 20–75 years)) and 119 patients (13.1%) who achieved pCR after NACT. In addition, 685 (75.7%) cases with HER2-low expression and 220 (24.3%) HER2-negative cases. We compared the clinicopathological characteristics and NACT efficacy of patients with different HER2 status (HER2-low group vs. HER2-negative group), and the results are displayed in **Table 1**. There were significant differences in stage_N (*P* = 0.014), histological grade (*P* = 0.001), ER status (*P* < 0.001), PgR status (*P* < 0.001), NACT regimens (*P* = 0.032) and NACT efficacy (*P* = 0.037) between patients with HER2-negative and HER2-low breast cancer. The patients with HER2-low breast cancer had a lower percentage of pCR compared to those with HER2-negative tumors.

**Table 1:** Baseline clinicopathological characteristics of breast cancer patients with different HER2 status.

Characteristic	Total (n= 905)	HER2-low (n= 685)	HER2-negative (n= 220)	*P* value^a^
Age (years)				0.105
< 45	496(54.8%)	365(53.2%)	131(59.5%)	
≥ 45	409(45.2%)	320(46.7%)	89(40.5%)	
Menopausal status				0.385
Premenopausal	315(34.8%)	233(34.0%)	82(37.3%)	
Perimenopausal	353(39.0%)	265(38.7%)	88(40.0%)	
Postmenopausal	237(26.2%)	187(27.3%)	50(22.7%)	
Stage_T				0.386
T1	88(9.7%)	71(10.4%)	17(7.7%)	
T2	653(72.2%)	487(71.1%)	166(75.5%)	
T3/T4	164(18.1%)	127(18.5%)	37(16.8%)	
Stage_N				**0.014**
cN0	324(35.8%)	234(34.1%)	90(40.9%)	
cN1	451(49.8%)	360(52.6%)	91(41.4%)	
cN2/cN3	130(14.4%)	91(13.3%)	39(17.7%)	
Histological grade				**0.001**
I/II	532(58.8%)	424(61.9%)	108(49.1%)	
III	373(41.2%)	261(38.1%)	112(50.9%)	
ER status				**< 0.001**
Negative	280(30.9%)	176(25.7%)	104(47.3%)	
Positive	625(69.1%)	509(74.3%)	116(52.7%)	
PgR status				**< 0.001**
Negative	395(43.6%)	270(39.4%)	125(56.8%)	
Positive	510(56.4%)	415(60.6%)	95(43.2%)	
Ki-67(%)				0.071
≤ 20	421(46.5%)	332(48.5%)	89(40.5%)	
(20, 50]	323(35.7%)	240(35.0%)	83(37.7%)	
>50	161(17.8%)	113(16.5%)	48(21.8%)	
NACT regimens				**0.032**
TEC	808(89.3%)	603(88.0%)	205(93.2%)	
EC-T	97(10.7%)	82(12.0%)	15(6.8%)	
NACT efficacy				**0.037**
pCR	119(13.1%)	81(11.8%)	38(17.3%)	
Non-pCR	786(86.9%)	604(88.2%)	182(82.7%)	

pCR, pathologic complete response; ER, estrogen receptor; PgR, progesterone receptor; HER2, human epidermal growth factor receptor2; NACT, neoadjuvant chemotherapy.

a *P* values were determined by chi-square tests. Bold values indicate statistical significance (*P* < 0.05).

### Analysis in different breast cancer subtypes

**Figure 2:**
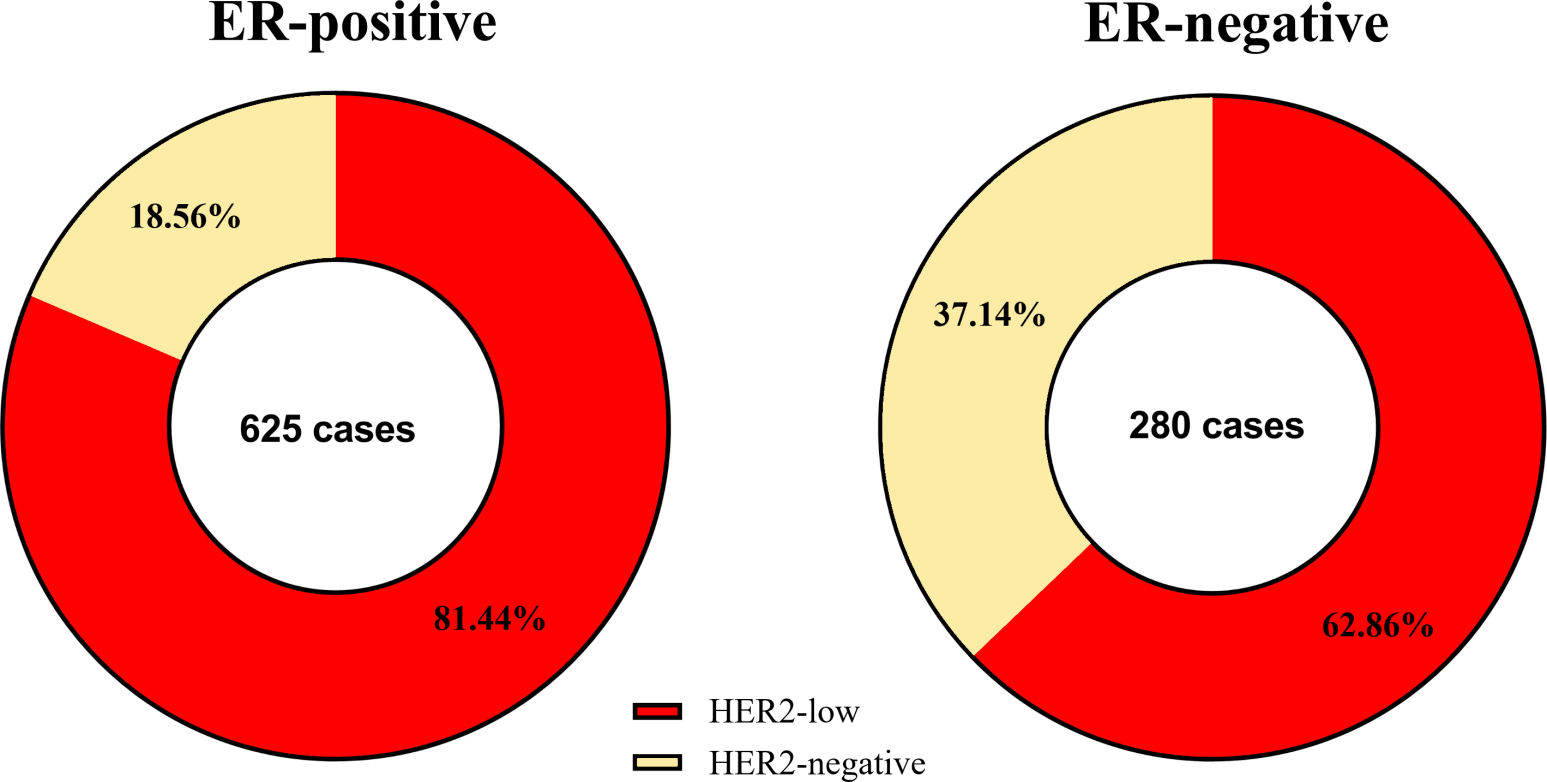
The compositions of different HER2 status by ER status.

**Figure 3:**
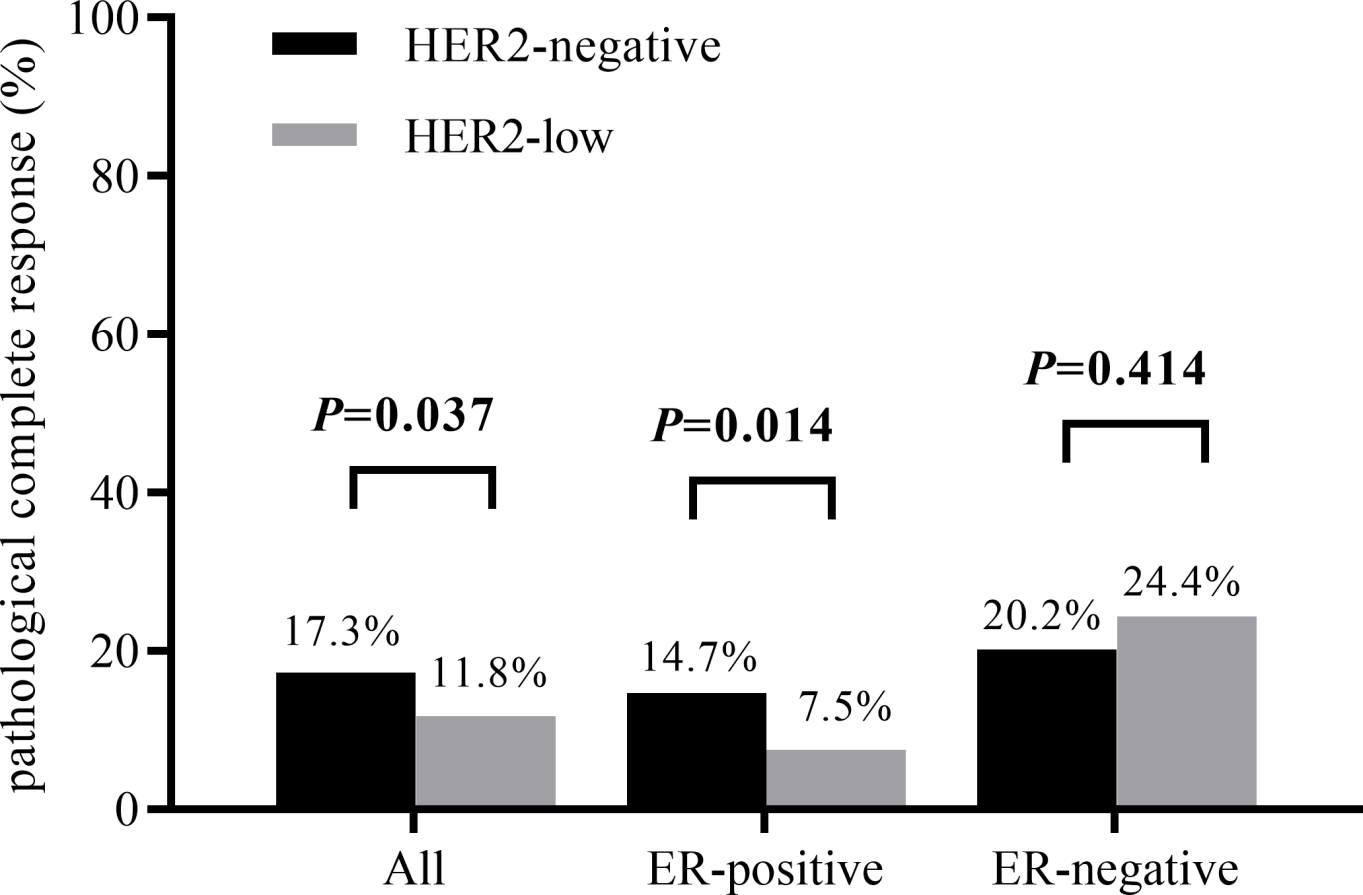
pCR rates according to HER2 status in different breast cancer subtypes (*P* value obtained by χ2 test).

The results of chi-squared test found that HER2 status was significantly associated with ER status. The distribution of different HER2 status in ER-positive patients and ER-negative patients was shown in **Figure 2**. Therefore, we analyzed the relationship between clinicopathological features and HER2 status in different breast cancer subtypes. In ER-positive breast cancer, there were significant differences in NACT efficacy (*P* = 0.014) and stage_N (*P* = 0.003) were significantly among HER2-low and HER2-negative breast cancers. The patients with HER2-low breast cancer had a lower percentage of pCR. Nevertheless, in ER-negative breast cancer only stage_N (*P* = 0.01) are related to HER2 status (**Table 2**). A significant association was observed between HER2 status and the probability to achieve a pCR. Of note, HER2-low breast cancer was associated with the low rate of pCR, especially in ER-positive patients, as shown in **Figure 3**. Here we found that there may exist some relevance between HER2 and HR, so subsequent analysis was performed in ER-positive patients and ER-negative patients respectively.

**Table 2:** Patient characteristics by HER2 status in different breast cancer subtypes.

Characteristic	ER-positive			ER-negative		
	HER2-low(n = 509)	HER2-negative(n = 116)	*P* value	HER2-low(n = 176)	HER2-negative(n = 104)	*P* value
Age (years)			0.139			0.379
< 45	273(53.6%)	71(61.2%)		92(52.3%)	60(57.7%)	
≥ 45	236(46.4%)	45(38.8%)		84(47.7%)	44(42.3%)	
Menopausal status			0.518			0.738
Premenopausal	171(33.6%)	45(38.8%)		62(35.2%)	37(35.6%)	
Perimenopausal	195(38.3%)	43(37.1%)		70(39.8%)	45(43.3%)	
Postmenopausal	143(28.1%)	28(24.1%)		44(25.0%)	22(21.2%)	
T stage			0.195			0.927
T1	58(11.4%)	8(6.9%)		13(7.4%)	9(8.7%)	
T2	359(70.5%)	91(78.4%)		128(72.7%)	75(72.1%)	
T3/T4	92(18.1%)	17(14.7%)		35(19.9%)	20(19.2%)	
N stage			**0.042**			**0.010**
cN0	142(27.9%)	45(38.8%)		91(51.7%)	39(37.5%)	
cN1	294(57.8%)	53(45.7%)		67(38.1%)	42(40.4%)	
cN2/cN3	73(14.3%)	18(15.5%)		18(10.2%)	23(22.1%)	
Histological grade			0.260			0.227
I/II	348(68.4%)	73(62.9%)		72(40.9%)	35(33.7%)	
III	161(31.6%)	43(37.1%)		104(59.1%)	69(66.3%)	
ER status			0.832			
(0, 10]	36(7.1%)	9(7.8%)				
(10, 40]	48(9.4%)	14(12.1%)				
(40, 70]	145(28.5%)	31(26.7%)				
>70	280(55.0%)	62(53.4%)				
PgR status			0.908			
Negative	103(20.0%)	27(23.3%)				
(0, 10]	75(14.7%)	16(13.8%)				
(10, 50]	98(19.3%)	22(19.0%)				
>50	233(45.8%)	51(44.0%)				
Ki-67(%)			0.715			0.072
≤ 20	273(53.6%)	67(57.8%)		57(32.4%)	21(20.2%)	
(20, 50]	176(34.6%)	36(31.0%)		62(35.2%)	47(45.2%)	
>50	60(11.8%)	13(11.2%)		57(32.4%)	36(34.6%)	
NACT regimens			0.098			0.102
TEC	452(88.8%)	109(94.0%)		151(85.5%)	96(92.3%)	
EC-T	57(11.2%)	7(6.0%)		25(14.2%)	8(7.7%)	
NACT efficacy			**0.014**			0.414
pCR	38(7.5%)	17(14.7%)		43(24.4%)	21(20.2%)	
Non-pCR	471(92.5%)	99(85.3%)		133(75.6%)	83(79.8%)	

Bold values indicate statistical significance (P < 0.05).

### Univariate and multivariate analysis on the factors of achieving a pCR after NACT

Based on univariate analysis, there were significant differences in stage_N (*P* = 0.029), histological grade (*P* = 0.003), ER (*P* < 0.001), PgR (*P* = 0.047), Ki-67 (*P* < 0.001) and HER2 status (*P* = 0.015) for achieving a pCR in patients with ER-positive breast cancer. Then, we included the factors (*P*< 0.05) in the multivariate analysis. We found that histological grade (*P* = 0.032), ER (*P* = 0.002), Ki-67 (*P* < 0.001) and HER2 status (*P* = 0.025) were independent predictors of achieving a pCR in ER-positive breast cancer (**Table 3**).

**Table 3:** Univariate and multivariate analysis of achieving a pCR in patients in ER-positive breast cancer.

Characteristics	Univariate analysis OR (95% CI)	*P* value	Multivariate analysis OR (95% CI)	*P* value
Age, years (≥ 45 vs < 45)	0.736 (0.417–1.300)	0.291		–
Menopausal status		0.207		–
Premenopausal	1 (reference)			–
Perimenopausal	0.625 (0.331–1.181)			–
Postmenopausal	0.577 (0.281–1.184)			–
T stage		0.229		–
T1	1 (reference)			–
T2	2.333 (0.704–7.735)			–
T3/T4	1.441 (0.360–5.777)			–
N stage		**0.029**		0.109
cN0	1 (reference)		1 (reference)	
cN1	0.579 (0.326–1.030)		0.668 (0.357–1.252)	
cN2/cN3	0.236 (0.069–0.803)		0.276 (0.076–1.000)	
Histological grade (III vs I/II)	2.322 (1.329–4.055)	**0.003**	1.952 (1.061–3.590)	**0.032**
ER status		**< 0.001**		**0.002**
(0, 10]	1 (reference)		1 (reference)	
(10, 40]	0.125 (0.033–0.472)		0.096 (0.023–0.411)	
(40, 70]	0.229 (0.100–0.528)		0.208 (0.076–0.571)	
>70	0.186 (0.086–0.400)		0.201 (0.076–0.532)	
PgR status		**0.047**		0.302
Negative	1 (reference)		1 (reference)	
(0, 10]	0.269 (0.088–0.818)		0.321 (0.096–1.071)	
(10, 50]	0.590 (0.268–1.297)		0.979 (0.393–2.440)	
>50	0.466 (0.241–0.902)		0.847 (0.370–1.937)	
Ki-67(%)		**< 0.001**		**< 0.001**
≤ 20	1 (reference)		1 (reference)	
(20, 50]	3.255 (1.659–6.388)		2.687 (1.317–5.484)	
>50	6.022 (2.760–13.138)		6.402 (2.804–14.617)	
NACT regimens (EC-T vs TEC)	0.481 (0.146–1.588)	0.230		–
HER2 status (HER2-low vs HER2-negative)	0.470 (0.255–0.866)	**0.015**	0.460 (0.233–0.906)	**0.025**

OR, odd ratio.

Bold values indicate statistical significance (P < 0.05).

Next, through the same analysis strategies, we found that stage_N (*P* = 0.001) and Ki-67 (*P* = 0.018) were independent predictors of achieving a pCR in ER-negative breast cancer (**Table 4**).

**Table 4:** Univariate and multivariate analysis of achieving a pCR in patients in ER-negative breast cancer.

Characteristics	Univariate analysis OR (95% CI)	*P* value	Multivariate analysis OR (95% CI)	*P* value
Age, years (≥ 45 vs < 45)	1.152 (0.659–2.015)	0.619		
Menopausal status		0.353		
Premenopausal	1 (reference)			
Perimenopausal	0.861 (0.463–1.604)			
Postmenopausal	0.562 (0.256–1.234)			
T stage		0.261		
T1	1 (reference)			
T2	0.871 (0.323–2.348)			
T3/T4	0.454 (0.137–1.508)			
N stage		**< 0.001**		**0.001**
cN0	1 (reference)		1 (reference)	
cN1	0.312 (0.162–0.600)		0.319 (0.164–0.622)	
cN2/cN3	0.271 (0.100–0.740)		0.276 (0.099–0.765)	
Histological grade (III vs I/II)	1.791 (0.974–3.291)	0.061	1.581 (0.834–2.996)	0.160
Ki-67(%)		**0.023**		**0.018**
≤ 20	1 (reference)		1 (reference)	
(20, 50]	3.047 (1.356–6.848)		2.733 (1.186–6.302)	
>50	2.667 (1.156–6.150)		2.554 (1.079–6.046)	
NACT regimens (EC-T vs TEC)	1.091 (0.466–2.554)	0.840		
HER2 status (HER2-low vs HER2-negative)	1.278 (0.709–2.304)	0.415		

Bold values indicate statistical significance (P < 0.05).

### Establish and validate the nomogram for NACT efficacy in ER-positive breast cancer

**Figure 4:**
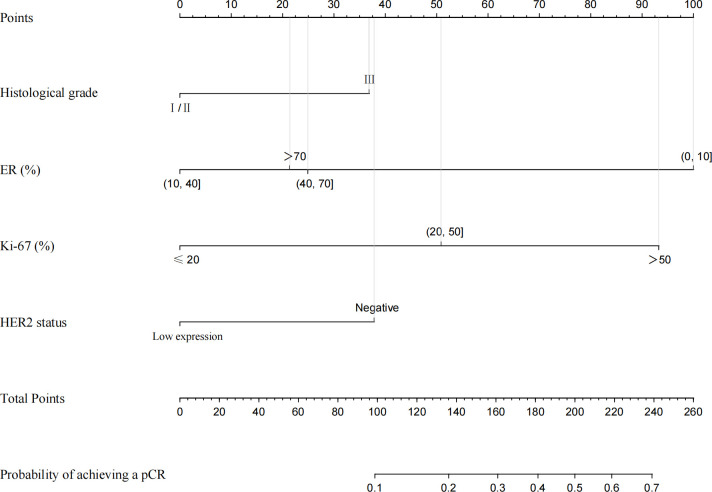
Nomogram for predicting pCR in ER−positive breast cancer patients after NACT. A line is drawn straight up to the point axis that corresponds with each patient variable to obtain the points. The sum of these points is located on the total score points axis. A line is drawn downwards to the risk axis to determine the possibility of achieving a pCR.

Through the univariate and multivariate logistic regression analysis, we established a nomogram to predict the probability of achieving a pCR after NACT in ER-positive breast cancer. The factors in the model included histological grade, ER expression, Ki-67 index and HER2 status (**Figure 4**).

**Figure 5:**
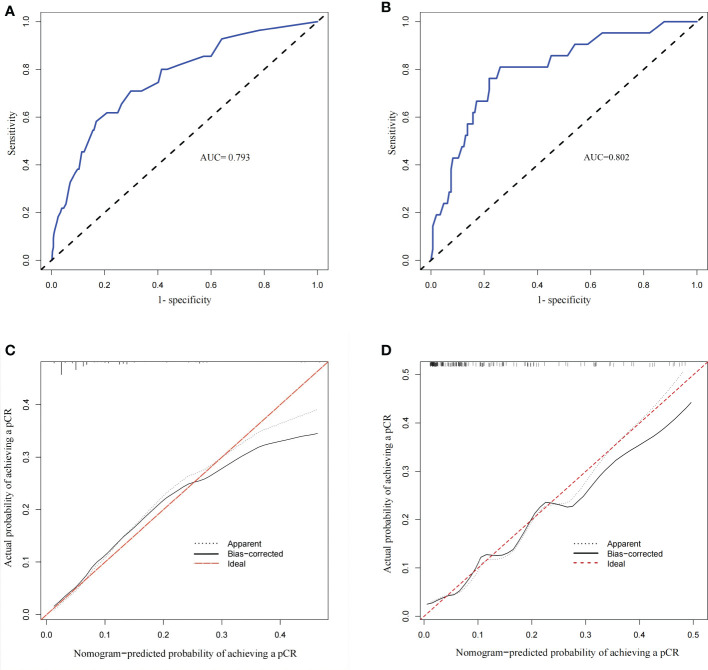
Calibration plots and Receiver operating characteristic (ROC) curves of the model. **(A)** ROC curve of the derivation group with an AUC of 0.795 (95% CI 0.735–0.855). **(B)** ROC curve of the validation group with an AUC of 0.802 (95% CI 0.785–0.819). **(C)** Calibration plot of the derivation group (The calibration plot depicts the calibration of the model in terms of the agreement between the predicted and the observed possibility of achieving a pCR in ER-positive breast cancer). **(D)** Calibration plot of the validation group.

According to this model, the ROC curve was drawn (**Figure 5A**), and the area under the curve (AUC) was 0.795 (95% CI: 0.735–0.855). The C-index of the prediction models was 0.787, which demonstrates good discriminative ability. The calibration plot revealed good agreement between the predictions and actual observations (**Figure 5C**).

Then, we took the data of patients diagnosed between 1 January 2020 and 31 June 2021 as external validation. There were no significant differences in age, menopausal status, stage_T, stage_N, histological grade, PgR status, HER2 status, Ki-67 index, NACT regimens and NACT efficacy between the derivation and validation groups (**Table 5**). Similarly, we established another nomogram through univariate and multivariate logistic regression analysis (**Supplement Figure 1**), these two models have good consistency. The ROC curve (AUC 0.802 (95% CI 0.785–0.819)) and calibration plot of validation group also indicated a good prediction ability(**Figures 5B, D**).

**Table 5:** Difference between the derivation and validation data groups.

Characteristic	Derivation Group(n = 625)	Validation Group(n = 167)	*P* value
Age (years)			0.670
< 45	344(55.0%)	95(56.9%)	
≥ 45	281(45.0%)	72(43.1%)	
Menopausal status			0.673
Premenopausal	216(34.6%)	61(36.5%)	
Perimenopausal	238(38.1%)	66(39.5%)	
Postmenopausal	171(27.4%)	40(24.0%)	
Stage_T			0.408
T1	66(10.6%)	12(7.2%)	
T2	450(72.0%)	123(73.7%)	
T3/T4	109(17.4%)	32(19.1%)	
Stage_N			0.149
cN0	187(29.9%)	61(36.5%)	
cN1	347(55.5%)	89(53.3%)	
cN2/cN3	90(14.9%)	17(10.2%)	
Histological grade			0.276
I/II	421(67.4%)	105(62.9%)	
III	204(32.6%)	62(37.1%)	
ER status			**0.035**
(0, 10]	45(7.2%)	15(9.0%)	
(10, 40]	62(9.9%)	27(16.2%)	
(40, 70]	176(28.2%)	52(31.1%)	
>70	342(54.7%)	73(43.7%)	
PgR status			0.251
Negative	130(20.8%)	35(21.0%)	
(0, 10]	91(14.6%)	34(20.4%)	
(10, 50]	120(19.2%)	33(19.8%)	
>50	284(45.4%)	65(38.9%)	
HER2 status			0.371
Negative	116(18.6%)	26(15.6%)	
Low	509(81.4%)	141(84.4%)	
Ki-67(%)			0.086
≤ 20	340(54.4%)	88(52.7%)	
(20, 50]	212(33.9%)	49(29.3%)	
>50	73(11.7%)	30(18.0%)	
NACT regimens			0.982
TEC	561(89.8%)	150(89.8%)	
EC-T	64(10.2%)	17(10.2%)	
NACT efficacy			0.141
pCR	55(8.8%)	21(12.6%)	
Non-pCR	570(91.2%)	146(87.4%)	

Bold values indicate statistical significance (P < 0.05).

In summary, these results showed that this nomogram has good efficacy in predicting the probability of achieving a pCR in ER-positive breast cancer.

## Discussion

HER2 is a prototype oncogene and its amplification represents a poor breast cancer subtype (17). Therapeutic interventions are focused on a small group of tumors that show an amplification of the HER2 gene with subsequent overexpression of the HER2 protein. High HER2 expression not only promotes the occurrence and development of tumors, but also is related to chemotherapy resistance (11). However, at present, in clinical the treatment strategy of patients with HER2-low breast cancer is the same as that of patients with HER2-negative breast cancer. In the present study, we retrospectively analyzed the clinicopathological features of patients with HER2-negative or HER2-low breast cancer and explored the influencing factors of achieving a pCR after NACT.

In our cohort of 905 breast cancer patients undergoing neoadjuvant chemotherapy, we found the intense association between HER2-low expression and HR-positive status (*P* < 0.001), and confirmed the possible role for ER in HER2-low expression biology (20, 21). Consequently, we performed a subgroup analysis of HER2 status in ER-positive and ER-negative breast cancer. Compared with ER-positive, HER2-negative breast cancer patients, a higher rate of axillary lymph node metastasis was found in patients with ER-positive, HER2-low breast cancer (*P* = 0.042). Analogous findings have been reported by previous studies, which consistently with our study have found a higher stage_N and lower histological grade in HER2-low breast cancer (22–24). In addition, it has also been found that HER2 status was related to age and stage_T in previous studies. Therefore, HER2-low breast cancer is different from HER2-negative breast cancer in clinicopathological features and may be recognized as a distinct diseases.

In the last century, the expression of HER2 was observed to confer resistance in breast cancer cells to several chemotherapy agents (25, 26). In the previous understanding HER2-low breast cancer was less malignant than HER2-positive breast cancer, however, there was no strong evidence that low expression of HER2 did not impact the process of tumorigenesis and drug resistance. In our study, overall in neoadjuvant chemotherapy for breast cancer patients, pCR rates were lower in HER2-low breast cancer patients (11.8%) than in HER2-negative patients (17.3%). Federica Miglietta et al. performed a retrospective study of 488 cases and obtained a consistent result that a lower proportion of pCR in HER2-low breast cancer patients (21.4%) after NACT than HER2-negative ones (33.6%) (27). Furthermore, another study including four prospective neoadjuvant clinical trials have reported analogous findings (pCR rates: 29.2% (HER2-low) vs 39.0% (HER2-negative)), which also showed that the proportion of pCR was significantly lower in HER2-low tumors versus HER2-negative tumors in the ER-positive subgroup (*P* = 0.024) but not in the ER-negative subgroup (*P* = 0.21).

Recently, a phase II study about a novel ADC reported promising preliminary results in HR-positive, HER2-low expression advanced breast cancer patients (16). Besides, trastuzumab-deruxtecan (T-Dxd) with a cleavable linkage to a potent topoisomerase I inhibitor payload and excellent membrane permeability, which laid the foundation for the treatment of HER2-low breast cancer (28). For instance, T-Dxd has achieved an objective response rate (ORR) of 37% in advanced breast cancer patients with low HER2 expression in preliminary trail (17). Recently, some encouraging results have been reported from the critical phase III trial DESTINY-Breast 04, which showed that regardless of HR status PFS and OS were both improved in HER2-low breast cancer patients treated with T-Dxd (29). Based on these promising results, several additional trails were gradually promoted. Interestingly, previous findings reported similar prognosis between HER2-low and HER2-negative breast cancer, so the drug used for the HER-low patients can better improve the prognosis of most breast cancer patients in the future (21, 30).

In addition to HER2 status, histological grade (*P* = 0.032), Ki-67 (*P* < 0.001), and ER status (*P* = 0.002) were independent predictors of achieving a pCR in ER-positive breast cancer *via* univariate and multivariate analysis. Based on these factors we developed an easy-to-use nomogram to predict the probability of achieving a pCR after NACT in ER-positive breast cancer patients. With this model we can rapidly predict the possibility of an ER-positive patient achieving a pCR after NACT. Then, similar analysis performed in patients with ER-negative breast cancer demonstrated that stage_N (*P* = 0.001) and Ki-67 (*P* < 0.018) were independent factors of achieving a pCR. The ER-negative breast cancer patient with earlier stage_N and higher Ki-67 index is more likely to achieving a pCR after NACT. Herein, patients with HER2-low breast cancers account for 75.7% of the total, thus, if the novel agents can be used in neoadjuvant therapy in the future, the pCR rate and the prognosis will be improved.

Our study has several limitations. Firstly, single institution and retrospective nature may responsible for both selection and information bias. Then, the lack of follow-up data has prevented us from conducting a deeper analysis of survival and recurrence rates in patients treated with neoadjuvant chemotherapy. However, our study collected detailed preoperative clinicopathological data and established a predictive model, which can better provide reference for clinical practice.

## Conclusion

For a long time, HER2-negative breast cancer and HER2-low breast cancer were recognized as the same biological subtype. Here, our study provides new insight into the clinicopathological features and NACT efficacy of HER2-low tumors. We evaluated some important factors that affect chemotherapy efficacy in a large cohort of patients undergoing neoadjuvant chemotherapy, with HER2 status being an independent influencing factor of pCR. Whereas, HER2-low breast cancer patients with a low probability of achieving a pCR will be candidates for new ADC drugs in the future.

## Data availability statement

The original contributions presented in the study are included in the article/**Supplementary Material**. Further inquiries can be directed to the corresponding author.

## Ethics statement

The studies involving human participants were reviewed and approved by Ethics Committee of the First Affiliated Hospital of Chongqing Medical University. Written informed consent for participation was not required for this study in accordance with the national legislation and the institutional requirements.

## Author contributions

(I) Conception and design: LT, ZL, and SL; (II) Administrative support: LT, SL; (III) Collection and assembly of data: ZL and LJ; (IV) Data analysis and interpretation: LT, LJ and YX; (V) Manuscript writing: LT, XS and ZL. All authors contributed to the article and approved the submitted version.
