# Supplementary figures and images for: Efficacy evaluation of neoadjuvant chemotherapy in patients with HER2-low expression breast cancer: A real-world retrospective study
Source: Front Oncol. 2022 Dec 19;12:999716. doi: 10.3389/fonc.2022.999716 (PMC9810386; doi:10.3389/fonc.2022.999716)

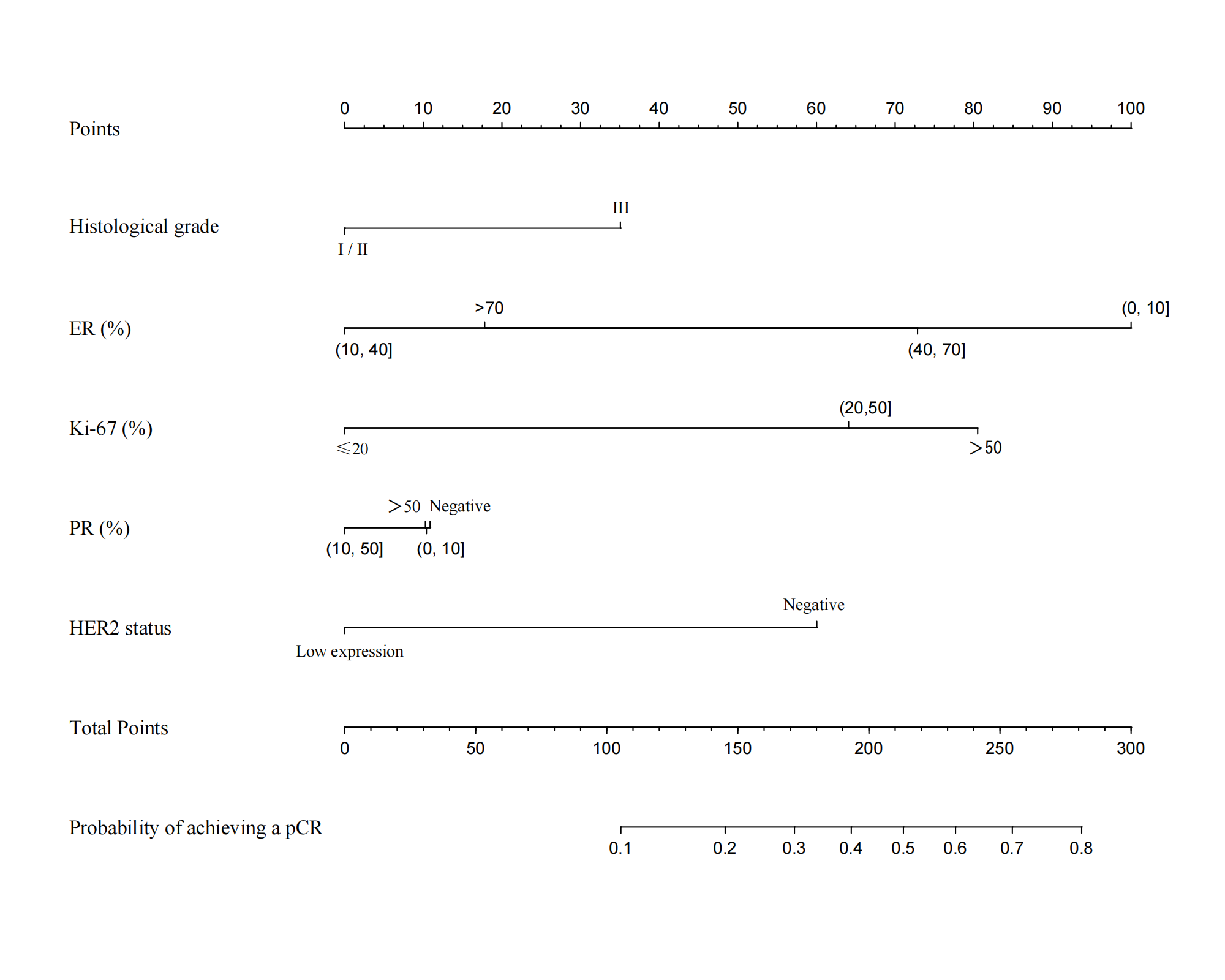

Supplement: Supplementary file 1 [file Image_1.tif]
